# Supplementary material for: Genome-Wide Analysis of the WRKY Transcription Factor Family in Roses and Their Putative Role in Defence Signalling in the Rose–Blackspot Interaction
Source: Plants (Basel). 2024 Apr 10;13(8):1066. doi: 10.3390/plants13081066 (PMC11054901; doi:10.3390/plants13081066)
Supplement: Supplementary file 1 [file plants-13-01066-s001.zip › Supplement_S3_Phylogenetic analysis of WRKYs of five rose species_new.pdf]

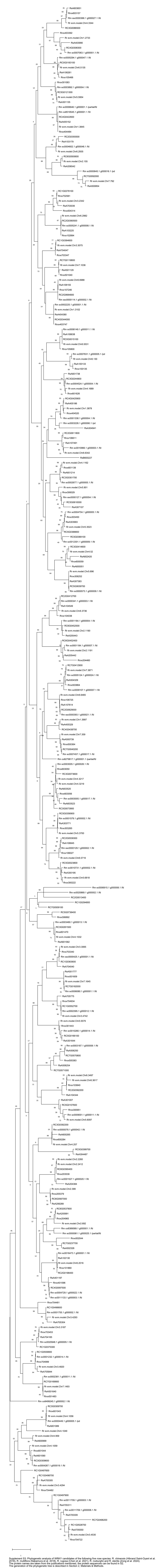

Supplement S3: Phylogenetic analysis of WRKY candidates of the following five rose species: R. chinensis (Hibrand Saint-Oyant et al. 2018), R. multiflora (Nakamura et al. 2018), R. rugosa (Chen et al. 2021), R. roxburghii and R. sterilis (Zong et al. 2024).
The protein names are taken from the publications mentioned, the protein sequences can be found in S2.
The generation of the phylogenetic tree is described in Section 2, Materials & Methods.
